# Natural extracts against agricultural pathogens: A case study of *Celtis australis* L.

**DOI:** 10.1002/fsn3.3325

**Published:** 2023-03-20

**Authors:** Tatjana Perović, Jovana Petrović, Uroš Gašić, Marina Kostić, Ana Ćirić

**Affiliations:** ^1^ Biotechnical Faculty, Centre for Subtropical Cultures University of Montenegro Bar Montenegro; ^2^ Department of Plant Physiology, Institute for Biological Research “Siniša Stanković”—National Institute of Republic of Serbia University of Belgrade Belgrade Serbia

**Keywords:** *Celtis australis* L., chemical characterization, cytotoxic, in vitro antifungal, methanol extracts

## Abstract

Plant extracts and other plant products have been used as an alternative to synthetic fungicides or an additional way to reduce their use. The choice of plant extracts and their application depends on their functional characteristics, availability, cost‐effectiveness, and their impact on phytopathogens, and also on the environment. Therefore, the present study aims to assess the potential of *Celtis australis* methanolic extracts as source of compounds with antifungal activity. Methanolic extracts prepared from leaves and unripe mesocarps of *C. australis* collected from different localities of Montenegro (Podgorica—PG, Donja Gorica—DG, and Bar—BR) were evaluated for their phenolic compounds' composition as well as antifungal and cytotoxic properties. Obtained results revealed that extracts contain various bioactive constituents including phenolic acids, flavonoids, and their derivatives. The predominant phenolic acid was ferulic acid, identified in leaf samples from DG (187.97 mg/100 g dw), while isoorientin was the most abundant phenolic compound found in all examined samples. Regarding antifungal potential of the tested samples, all but one (prepared from mesocarp BR) possessed higher activity than Previcur, a commercial systemic fungicide intended to control seedlings. In vitro studies on HaCaT cell line showed that the extracts had no toxic effect toward the tested cell line. These results lead to the conclusion that methanolic extracts of *C. australis* can become an alternative to the use of synthetic fungicides in agriculture. Those extracts represent natural biodegradable fungicides and enable more efficient control of pathogenic fungi.

## Graphical abstract

Our investigation has shown chemically characterized methanol extracts of leaves and unripe mesocarps of *Celtis australis*, and evaluated their antifungal and cytotoxic potential. Those extracts represent natural biodegradable fungicides and enable more efficient control of pathogenic fungi.

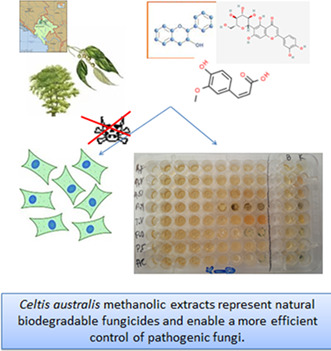

## 1 INTRODUCTION

*Celtis australis* L. (Cannabaceae) has wide distribution throughout mild and warm climate zone—from southern Europe and northern Africa, through southwestern Asia, to India. This Mediterranean deciduous tree may reach monumental size and longevity: it may grow up to 25 m in height, 10 m in width, and live as long as 1000 years (Ota et al., 2017). Huge stumps are common in town alleys or around village houses in Montenegro and Croatia (Ota et al., 2017). It is commonly referred to as the European nettle tree, Mediterranean hackberry, or lote tree. As for the appearance, the leaves are asymmetrical, slightly reminiscent of a nettle leaf—hence its common name; fruits (spherical drupes, 9–12 mm long) are green in middle of the year, reaching ripening phase in autumn.

Both leaves and fruits have been traditionally used as a natural remedy for the preparation of decocts with healing properties; treatment of gastrointestinal issues (diarrhea, dysentery, colic, and peptic ulcers), as well as regular/irregular periods in reproductive‐age women, has been usually mentioned (Chevallier, 1996). Mentioned activities may be related to the chemical composition of *C. australis*. Previously published phytochemical analyses revealed that Mediterranean hackberry contains rare flavonoids: flavone *O*‐glycoside and flavone *C*‐glycosides, as well as tannins and saponins (Spitaler et al., 2009; Zehrmann et al., 2010). Furthermore, ripe fruits have high content of sugars, proteins, crude fibers, oils, ash, and minerals (Demir et al., 2002). A significant quantity of lipophilic compounds were also determined in fruits of wild‐growing *C. australis* from Iran, such as linoleic acid, *γ*‐tocopherol (87%), and *β*‐sitosterol (Nodeh et al., 2020). According to Badoni et al. (2010), ripe fruits are rich in celtisanin (sulfonated phenolic compound) along with several fatty acids, methyl oleate, and methyl palmitate as the main compounds. This was confirmed in several earlier reports (Badoni et al., 2010; El‐Alfy et al., 2011; Filali‐Ansari et al., 2015; Ota et al., 2017; Shokrzadeh et al., 2019). According to the published data, extracts prepared from fruit mesocarp and leaves present valuable sources of several groups of compounds with antimicrobial, antioxidant, and cytotoxic potential.

Pathogenic fungi are responsible for as many as 100,000 diseases in plants leading to reduced yield in crops, and medicinal and other plants used as food (Agrios, 2005). Control of their growth is most often accomplished with several available chemical agents that may have a profound effect on the environment and health of people, consummating treated plants as food. Furthermore, their use has one more main disadvantage–after a waiting period, the residues of their degradation enter the food chain and can lead to serious health deleterious effects on consumers (Chen et al., 2008; Scordino et al., 2008). Moreover, their excessive and inadequate use may induce acquired resistance in fungi.

Along with this emerging health‐related problem, accompanying economic losses additionally encourage the scientific community to search for novel, safer ways to control growth of these fungal organisms. Recently, there has been a growing interest in natural products as antifungal agents due to their availability, fewer side effects, and/or toxicity, as well as the fact they are “environment‐friendly.” With regard to this, new plant‐based products may serve as a promising alternative in controlling these phytopathogens. Since the species is used in traditional medicine to treat gastrointestinal ailments, related to pathogenic microbes, we strived to repurpose the use and investigate the extracts of this species against agro‐industrial pathogens. Furthermore, the present study chemically characterized methanol extracts of leaves and unripe mesocarps of *C. australis*, and evaluated their antifungal and cytotoxic potential.

## 2 MATERIALS AND METHODS

### 2.1 Reagents

Solvents for analyses (acetonitrile and formic acid) were of LC–MS grade (Fisher Scientific). Methanol and ethanol (HPLC grade) were purchased from AppliChem. Ultrapure water was generated by a deionization (Millipore) system. Analytical standards of phenolic compounds (gallic acid, protocatechuic acid, gallocatechin, syringic acid, epigallocatechin, aesculetin, caffeic acid, isoorientin, rutin, vitexin, isoquercetin, ferulic acid, astragalin, apigenin, luteolin, apigenin, naringenin, and hispidulin) supplied by Sigma‐Aldrich were from 95% to 99% purity.

### 2.2 Material collection and preparation of the extract

Leaves and unripe mesocarps of *C. australis* L. were collected from three localities: Bar (BR) (42°06′04″N; 19°06′09.8″E), Donja Gorica (DG) (42°25′31″N; 19°12′41″E), and Podgorica (PG) (42°26′33″N; 19°14′34″E), Montenegro, in June 2019 and air dried for 5 days at 25°C. Subsequently, they were ground to a fine powder and used for extraction. Briefly, 10 g of sample powder was mixed with 240 mL of methanol, and left overnight at −20°C. The resulting mixture was sonicated for 15 min, then centrifuged at 4000 *g* for 10 min at +4°C, and subsequently filtered through Whatman No. 4 paper (Vaz et al., 2010). The residue was re‐extracted with three additional portions of methanol (3 × 100 mL) following the same procedure (ultrasonic bath and filter paper). The combined extract was evaporated at 40°C (rotary evaporator Büchi R‐210) to dryness. Prior to analyses, extract was dissolved in appropriate solvent.

### 2.3 LC–MS analysis of phenolic compounds

Quantification of phenolic compounds in methanolic extracts of *C. australis* was performed using LC–MS, previously described by Gašić et al. (2015). A stock solution of a mixture of phenolic standards with a concentration of 1000 mg/L was prepared in methanol. By diluting the stock solution with the mobile phase (H_2_O:ACN = 1:1, v/v), working solutions were obtained in concentrations of 0.025, 0.050, 0.100, 0.250, 0.500, 0.750, and 1000 mg/L. Separation of the phenolic compounds in the samples was achieved with Dionex Ultimate 3000 UHPLC system equipped with a diode array detector (DAD) and TSQ Quantum Access Max triple‐quadrupole (QqQ) mass spectrometer (MS), obtained by Thermo Fisher Scientific (Basel, Switzerland). Specification of the chromatographic parameters (analytical column, mobile phases, and gradient elution program) and MS settings was as previously described by Gašić et al. (2015). Phenolic compounds were identified and quantified by comparison with commercial standards, and their concentration was expressed as mg/100 g dry weight (dw).

### 2.4 In vitro antifungal assay

Antifungal activity of *C. australis* methanolic extracts was determined by the modified microdilution method (CLSI, 2009; Tsukatani et al., 2012). The following fungal strains were used: *Aspergillus flavus* (ATCC 9170), *Aspergillus niger* (ATCC 6275), *Aspergillus terreus* (ATCC 16792), *Penicillium ochrochloron* (ATCC 9112), *Penicillium verrucosum* var. *cyclopium* (food isolate), and *Fusarium verticillioides* (strawberry isolate). All the tested microorganisms are deposited at the Mycological Laboratory, Department of Plant Physiology, Institute for Biological Research “Siniša Stankovic”—National Institute of Republic of Serbia, University of Belgrade, Serbia. Prior to the antifungal assay, fungi were cultured for 14 days on solid malt agar at 25°C, after which the spore inoculum was prepared. Spores were washed using sterile saline and 0.1% Tween 80 (v/v), and the suspension was adjusted to a concentration of approximately 1.0 × 10^5^ CFU in a final volume of 100 μL per well by microscopic enumeration with a cell‐counting hemotocytometer (Neubauer chamber; Paul Marienfeld). Obtained results were presented as minimal inhibitory (MICs) and minimal fungicidal concentrations (MFCs) needed to effectively retard fungal growth. Commercially available antifungal agents ketoconazole (Zorkapharma) and Previcur energy (Bayer Crop Science) were used as positive controls. Ethanol solution (30%) was used as negative control. Experiments were repeated twice.

### 2.5 Crystal violet test with human keratinocytes (HaCaT cell line)

Crystal violet assay was used to evaluate cytotoxic effect of the tested samples, according to the previously described protocol by Stojkovic et al. (2020) with some modifications. The spontaneously immortalized human skin keratinocytes (HaCaT) cell line used for the assay was grown in high‐glucose Dulbecco's modified Eagle medium (DMEM) supplemented with 10% fetal bovine serum (FBS), 2 mM L‐glutamine, and 1% penicillin and streptomycin (Invitrogen) at 37°C in 5% CO_2_. Twenty‐four hours prior to the treatment with extracts, 1 × 10^4^ cells/well were seeded in a 96‐well plate. Potassium dichromate (K_2_Cr_2_O_7_) was used as a positive control and PBS as a negative one. The experiment was performed in triplicate. The absorbance of the resulting solution was measured using microplate reader at 590 nm (OD_590_). The results were expressed as IC_50_ (%) value in μg mL^−1^. The following criterion was used to categorize cytotoxic activity of extract to HaCaT cell line: IC_50_ ≤ 20 μg mL^−1^ = highly cytotoxic, IC_50_ ranged between 21 and 250 μg mL^−1^ = moderately cytotoxic, IC_50_ ranged between 201 and 500 μg mL^−1^ = weakly cytotoxic, and IC_50_ > 501 μg mL^−1^ = no cytotoxicity.

## 3 RESULTS

### 3.1 Phenolic compounds

LC–MS analysis of the tested methanolic extracts, from three localities, showed the presence of several bioactive compounds including phenolic acids, flavonoids, and their derivatives (Table 1). The individual phenolic acids were identified in Mediterranean hackberry samples: hydroxybenzoic acids (protocatechuic, gallic, and syringic acid) and hydroxycinnamic acids (caffeic and ferulic acid, and aesculin). Syringic acid, ferulic acid, and aesculin were detected in all tested samples, while protocatechuic and gallic acid among hydroxybenzoic and caffeic acid were not identified in mesocarp extracts from localities BR and DG. The predominant phenolic acid was ferulic acid found in leave samples from DG (187.97 mg/100 g dw). In the tested samples, 12 flavonoids were quantified: flavan‐3‐ols (gallocatechin, epigallocatechin, and rutin), flavone *C*‐glycosides (isoorientin, vitexin, apigetrin, luteolin, apigenin, and hispidulin), flavonol 3‐*O*‐glucoside (astragalin and isoquercetin), and flavanone—naringenin. Rutin was detected in all the tested extracts, whereas the presence of gallocatechin and epigallocatechin was not confirmed in both extracts (leaves and mesocarp) from the locality of Bar.

**TABLE 1:** Concentration (mg/100 g dw) of phenolic compounds identified in methanol extracts from mesocarp and leaves of *C. australis*, collected in localities across Montenegro.

mg/100 g	Mesocarp (BR)	Mesocarp (DG)	Mesocarp (PG)	Leaves (BR)	Leaves (DG)	Leaves (PG)
Gallic acid	ND	ND	56.14	29.86	57.91	96.51
Protocatechuic acid	ND	ND	44.25	27.65	39.48	51.93
Gallocatechin	ND	ND	663.47	ND	1007.51	28.72
Syringic acid	17.09	35.08	69.14	50.29	70.75	69.59
Epigallocatechin	ND	21.08	62.04	ND	66.15	ND
Aesculetin	5.77	17.61	24.66	34.72	23.37	15.15
Caffeic acid	ND	ND	13.64	5.50	6.85	5.31
Isoorientin	36.40	293.46	1362.66	1117.54	2316.05	2026.90
Rutin	3.76	8.33	73.69	16.63	119.29	73.95
Vitexin	4.08	35.02	445.29	130.22	734.01	663.09
Isoquercetin	ND	ND	17.12	ND	23.26	20.59
Ferulic acid	15.92	43.09	162.61	114.12	187.97	114.78
Astragalin	0.21	5.16	26.42	59.86	53.56	40.40
Apigetrin	ND	1.78	ND	2.19	ND	ND
Luteolin	ND	ND	14.09	6.83	6.82	9.43
Apigenin	2.97	2.98	3.63	4.01	3.12	3.57
Naringenin	ND	ND	4.34	5.23	ND	ND
Hispidulin	2.10	2.12	2.67	2.87	2.36	2.61

*Note*: Localities: (BR)—Bar; (DG)—Donja Gorica; (PG)—Podgorica.

Abbreviation: ND, not detected.

Abundance of the compounds depended on the locality of the sample, as well as the tissue type from which the extract was prepared. Thus, several compounds were identified in leaves collected from all three localities, but not in all samples of fruit mesocarp, such as gallic acid, protocatechuic and caffeic acid, and luteolin (Table 1). Nevertheless, the presence of syringic acid, aesculetin, isoorientin, rutin, vitexin, ferulic acid, astragalin, apigenin, and hispidulin was confirmed in all leaves and mesocarp samples. The amount of the compounds varied significantly; the concentration of isoorientin, vitexin, apigenin, and hispidulin was in range 2.12–2316.05 mg/100 g dw. Astragalin was quantified in all the tested samples in wide range, with BR locality mesocarp and leaves sample as the richest source of this compound, 0.21 mg/100 g dw and 59.86 mg/100 g, respectively. The most abundant phenolic compound turned out to be isoorientin, with the highest detected amount among all the quantified compounds–2316.05 mg/100 g dw (extract prepared from leaves of *C. australis* collected in DG locality). As for the flavonoids, gallocatechin was detected in significant amount in this sample as well (1007.51 mg/100 g dw).

### 3.2 Antifungal activity of *C. australis* methanolic extracts

Regarding results of antifungal activity (Table 2), obtained data indicate that most of the evaluated extracts possessed inhibitory activity toward tested strains, except the sample prepared from mesocarp from the BR locality, which did not affect fungal growth in concentrations up to 8 mg mL^−1^. Inhibitory concentrations ranged between 1.00 and 4.00 mg mL^−1^, whereas fungicidal activities were in the range 2.00–8.00 mg mL^−1^. The tested extracts efficiently inhibited fungal growth (MIC 1.00–4.00 mg mL^−1^ and MFC 2.00–8.00 mg mL^−1^) in comparison to commercial fungicide Previcur, used as a positive control (MIC 25.0–75.0 mg mL^−1^ and MFC 50.0–200.0 mg mL^−1^). As opposed to this, the antifungal potential of the tested samples was lower than the other antifungal agent Ketoconazole (MIC 0.20–1.50 mg mL^−1^ and MFC 0.50–2.00 mg mL^−1^), also used as a positive control. Samples from all three localities, prepared from both mesocarp and leaves showed rather uniform and moderate antifungal activity, much better than Previcur, yet significantly lower than Ketoconazole.

**TABLE 2:** Antifungal activity of *Celtis australis* methanolic extracts (mesocarp and leaves) from three localities in Montenegro (mg mL^−1^).

	*Aspergillus flavus*		*Aspergillus niger*		*Aspergillus terreus*		*Penicillium ochlochloron*		*Penicillium verrucosum* var. *cyclopium*		*Fusarium verticillioides*	
	MIC	MFC	MIC	MFC	MIC	MFC	MIC	MFC	MIC	MFC	MIC	MFC
Mesocarp (BR)	*	*	*	*	*	*	*	*	*	*	*	*
Mesocarp (DG)	2.00	4.00	4.00	8.00	1.00	2.00	1.00	2.00	2.00	4.00	4.00	8.00
Mesocarp (PG)	2.00	4.00	2.00	4.00	1.00	2.00	1.00	2.00	1.00	2.00	2.00	4.00
Leaves (BR)	2.00	4.00	2.00	4.00	1.00	2.00	1.00	2.00	1.00	2.00	2.00	4.00
Leaves (DG)	2.00	4.00	1.00	2.00	1.00	2.00	1.00	2.00	1.00	2.00	2.00	4.00
Leaves (PG)	2.00	4.00	1.00	2.00	1.00	2.00	1.00	2.00	1.00	2.00	2.00	4.00
Previcur	50.0	100.0	75.0	200.0	25.0	75.0	25.0	50.0	50.0	100.0	50.0	100.0
Ketoconazole	1.50	2.00	0.20	0.50	0.10	0.20	0.20	0.50	0.20	0.30	0.20	0.50

*Note*: Localities: (BR)—Bar; (DG)—Donja Gorica; (PG)—Podgorica.

* Not active at tested concentrations of up to 8 mg mL^−1^.

### 3.3 Cytotoxic potential of *C. australis* methanolic extracts

Due to pressure from societies for the prevention of cruelty to animals to reduce the number of animal testing, there is a strong need to develop new model systems to evaluate adverse effects of the compounds on the skin. Use of HaCaT keratinocyte cell line from adult human skin may be considered a suitable alternative and a reliable in vitro model for preliminary screening of various compounds, more precisely their skin irritability features.

Obtained results of this study (Table 3) showed that IC_50_ values for all the tested samples were higher than 500 μg mL^−1^, thus indicating lack of cytotoxic activity.

**TABLE 3:** Cytotoxic properties of *Celtis australis* methanolic extracts (mesocarp and leaves) from three localities in Montenegro on human immortalized keratinocyte cell line.

Samples	IC_50_ (μg mL^−1^) HaCaT cell line
Mesocarp (BR)	>500
Mesocarp (DG)	>500
Mesocarp (PG)	>500
Leaves (BR)	>500
Leaves (DG)	>500
Leaves (PG)	>500
K_2_Cr_2_O_7_	16.29 ± 1.42

*Note*: Localities: (BR)—Bar; (DG)—Donja Gorica; (PG)—Podgorica toward the used HaCaT cell line.

## 4 DISCUSSION

Previous phytochemical studies revealed that *C. australis* leaves are a rich source of flavonoid compounds. According to Spitaler et al. (2009), leaves collected from Italy are a rich source of flavone *C*‐glycosides (acacetin 7‐*O*‐glucoside, isovitexin, and cytisoside), whereas the presence of a new flavonoid compound, namely *C*‐glycoside 8‐(4‐α‐rhamnosyl‐2″‐*O*‐*β*‐d‐galactopyranosylvitexin), has been demonstrated in leaves of *C. australis* collected from Egypt (El‐Alfy et al., 2011). Three known flavonoids apigenin, quercetin, and quercetin glucosides, four triterpenoids, and one steroid have been isolated from fresh bark and mesocarps of *C. australis* from India (Semwal & Semwal, 2013). A sample collected in Croatia, not far from Montenegro, contained epicatechin, gallic acid, vanillic acid, 3,4‐dihydroxybenzaldehyde, delphinidin‐3,5‐di‐*O*‐glucoside, cyanidin‐3,5‐di‐*O*‐glucoside, and pelargonidin‐3,5‐di‐*O*‐glucoside (Ota et al., 2017). According to Ota et al. (2017), aqueous and ethanolic extracts of *C. australis* fruit and leaves collected at different growth stages also contained gallic acid and epicatechin in *C. australis* leaves, which is in accordance with results obtained in this study (Table 1).

As for the rest of the detected phenolic compounds in this study, protocatechuic acid, syringic acid, hydroxycinnamic acids, rutin, isoorientin, apigetrin, luteolin, hispidulin, flavonol 3‐*O*‐glucosides, and naringenin (Table 1), it may be concluded that they have been detected in *C. australis* samples for the first time. Our phytochemical screening indicates difference in chemical composition of leaves and mesocap, with leaves containing higher amount of phenolic compounds than mesocarp. Furthermore, all three prepared extracts are a rich source of flavonoids as well, especially isoorientin, vitexin, rutin, and gallocatechin. Their concentration is dependent on the climatic characteristics (particularly temperature), as well as the tested plant part of Mediterranean hackberry. Discrepancy was also observed with respect to the amount of phenolic compounds among samples collected from different localities: the highest amount was detected in leaves collected in Donja Gorica, followed by the sample collected in Podgorica and Bar. As for the content of phenolic compounds in mesocarps, the following descending order was observed: Podgorica > Donja Gorica > Bar. Even though the distance between the two localities Podgorica and Donja Gorica is about 10 km, and another two positions Podgorica and Bar is 50 km, the quantity of the phenolics significantly varied among the populations due to different impacts of environmental factors.

Regarding antimicrobial potential of *C. australis*, several studies demonstrated that various hackberry extracts, prepared with different solvents, may act as antibacterial agents (Ahmad et al., 2012; Alonso‐Esteban et al., 2018; Nodeh et al., 2020; Ota et al., 2017; Zehrmann et al., 2010). Nevertheless, antifungal properties of *C. australis* extracts were poorly investigated (Alonso‐Esteban et al., 2018; Nodeh et al., 2020; Ota et al., 2017). Ota et al. (2017) showed that ethanolic extracts prepared from leaves harvested at the end of October were active against *Candida albicans*, *C. parapsilosis*, and *Rhodotorula mucilaginosa*. Furthermore, methanolic aqueous extract (80:20, v/v) of *C. australis* seed exhibited antifungal activity toward six micromycetes, with *Aspergillus ochraceus* and *Penicillium funiculosum* as the most susceptible to the activity of the tested extracts (Alonso‐Esteban et al., 2018). According to the study by Filali‐Ansari et al. (2015), antifungal effects against *C. albicans*, *C. tropicalis*, and *A. niger* have been shown with ethyl acetate and hydromethanolic extracts of leaves and seeds from *C. australis*.

Results obtained in the present study (aside from the BR mesocarp extract) indicate rather uniform and moderate antifungal activity toward the tested fungal strains (Table 2), which was also observed in the study by Alonso‐Esteban et al. (2018). Lower amount of phenolic compounds in BR mesocarp extract may be the underlying reason for the weaker antifungal potential of this sample toward all the tested micromycetes. Along with this, specific combination of flavones and flavanones in different proportions may be responsible for better antifungal activity. Moreover, since the presence of flavonoids in natural matrices has been associated with several medicinal (including antimicrobial) properties, extracts prepared from leaves collected in Donja Gorica with high amounts of flavonoids (especially isoorientin and gallocatechin) may have the potential to be used as antimicrobial agent (Dinda et al., 2006).

Our study indicated that the extracts were not cytotoxic to HaCaT cell line, indicating that the extracts could be considered safe for use. These findings point to agro‐industrial application of the extracts to control growth of pathogenic microbes, without possible side effects on human health, which is of prime importance. The plant extracts are of natural origin with antimicrobial activity against wide range of pathogens and without toxicity to human skin cells, which makes them environmentally friendly and their use seems to be sustainable.

Previous reports on *C. australis* cytotoxic potential claimed promising activity of crude extracts toward several cancer cell lines: hepatocellular carcinoma (HEP‐G2), gastric carcinoma (NCI‐N87), leukemia carcinoma (CCRF‐CEM), human colon carcinoma (COLO 205), and ovary adenocarcinoma (NIH:OVCAR‐3) (El‐Alfy et al., 2011). dos Santos Branco et al. (2015), proved that *C. angustifolia*, rich in flavonoids and tannins among other compounds, strongly inhibited growth of Hep490 cell line (larynx carcinoma). Moreover, mentioned study showed evidence that cancerous cells were more susceptible to the activity of extract than non‐cancerous epithelial cells HEK‐293, which has been attributed to the inhibitory activity of polyphenols. Possible underlying mechanism may have included increased production of reactive oxygen species (ROS) which was followed by apoptosis.

The present study, which was conducted to evaluate the chemical, cytotoxic, and antifungal potential of *C. australis*, has shown that another plant may provide an alternative to the use of synthetic fungicides in agriculture. These extracts support natural, biodegradable fungicides and allow more efficient control of pathogenic fungi and minimization of their negative impact on biodiversity and human health. This may be supported by data published by Vondráková et al. (2020) which demonstrate that phenolic compounds play an important role in plant defense mechanism against biotic and abiotic stress, particularly against phytopathogenic fungi (Lattanzio et al., 2006; Vagiri et al., 2017). Presented data indicate that all the tested extracts are a rich source of phenolic compounds and flavonoids. Moreover, they demonstrate that concentration and presence of certain compounds are dependent on the tissue type as well as the locality of the plant—that is, geographic and climatic conditions of the region where the plant was collected. Nevertheless, they do not have a profound influence on biological activity, since the obtained results regarding antifungal and cytotoxic activities turned out to be rather uniform, regardless of the origin of the plant.

In recent decades, significant effort has been made to find alternative ways to protect crops in agriculture since use of many agents was restricted due to their adverse effects on both human health and the environment (Card et al., 2016). This turned out to be not an easy task after all since along with efficiency, the potential new agent has to be safe for use and environmentally sustainable. Natural matrices proved to be more than an adequate alternative to the used chemical agents, which is supported by data obtained in the present study as well. Flavonols (quercetin), flavones (luteolin, apigenin), and their glycosylated derivatives (quercitrin, isoquercitrin, rutin, and apigetrin) identified in the tested hackberry extracts turned out to be promising group of substances with already demonstrated low systemic toxicity (Ivanov et al., 2021), good antifungal potential (much better activity than widely used Previcur), and low cytotoxic properties. The obtained results should be further evaluated in order to expand/increase utilization of wild‐growing *C. australis* and possibly develop new agricultural and/or medicinal agents. This is a matter of highest priority since it is difficult to develop novel fungicides that encompass broad‐spectrum effectiveness, enhanced bioavailability, and low toxicity—a case obtained with *C. australis* extracts.

## 5 CONCLUSIONS

This study showed that methanolic extracts of *C. australis* represent an abundant source of bioactive substances, mainly phenolic acids and flavonoids. Moreover, the results of the study demonstrated that the quantity of compounds differs more within parts of the same plant than among populations. As for the bioactivity assays, extracts successfully inhibited growth of the tested pathogenic fungi and showed low toxicity on cell line from adult human skin. All the presented results indicate that Mediterranean hackberry may have a significant role in the discovery of novel prototype therapeutic agents. In summary, this study offers the possibility of developing strategies for controlling pathogenic fungi with plant extracts, as natural biodegradable fungicides. Also, the presented research corresponds to the current requirements of agricultural measures because it provides safe and eco‐friendly protection of plant species that are important for human use.

## FUNDING INFORMATION

The authors acknowledge the financial support of the Ministry of Education, Science and Technological Development of the Republic of Serbia (Grant No. 451‐03‐02263/2018‐09/26) and Ministry of Science, Technological Development and Innovation (Grant No. 451‐03‐47/2023‐01/200007).

## ACKNOWLEDGEMENTS

The authors are grateful to all participants of bilateral project between R. Serbia and Montenegro, title: “Biocontrol of phytopathogenic fungus by natural products from subtropic plants order Rosales”.

## CONFLICT OF INTEREST STATEMENT

The authors declare that there are no conflicts of interest.

## Data Availability

Data are openly available in a public repository that issues datasets with DOIs.
